# Dr. Philip Heidrich

**Published:** 1914-07

**Authors:** 


Dr. Philip Heldrich, University of Maryland, 1883, died at his home in Baltimore, May 1. He was formerly a resident of Elmira.

